# Supplementary material for: Allele Frequencies and Forensic Data of 25 STR Markers for Individuals in Northeast Brazil
Source: Genes (Basel). 2023 May 29;14(6):1185. doi: 10.3390/genes14061185 (PMC10298256; doi:10.3390/genes14061185)
Supplement: Supplementary file 1 [file genes-14-01185-s001.zip › genes-2403159-supplementary.pdf]

Supplementary Material S1

Table S1. Characterization of allelic variation in Bahia population.

Range of alleles		Number of alleles	The most frequent alleles		The less frequent alleles	
			Allele	Frequency	Allele	Frequency
CD4	5 to 15	12	5	0.328	10.4	0.001
			10	0.206	7	0.007
D8S639	11 to 34.3	19	27	0.208	11	0.001
			28	0.191	16	0.001
PENTA E	2.2 to 23	19	12	0.132	2.2	0.001
			7	0.122	23	0.001
PENTA D	2.2 to 26	16	10	0.171	6	0.001
			9	0.164	24	0.001
D3S1358	13 to 19	7	16	0.290	13	0.007
			15	0.280	19	0.005
VWA31	11 to 21	10	16	0.253	11	0.008
			17	0.233	21	0.001
D16S539	5 to 15	9	11	0.319	5	0.001
			12	0.236	15	0.004
CSF1PO	6 to 14	9	12	0.319	6	0.001
			10	0.273	14	0.012
TPOX	6 to 12	7	8	0.396	7	0.004
			11	0.276	12	0.047
D8S1179	8 to 19	12	14	0.279	19	0.001
			13	0.255	18	0.001
D21S11	24.3 to 37	21	30	0.233	25.2	0.001
			29	0.201	26	0.001
D18S51	9 to 25	19	15	0.158	9	0.001
			16	0.154	13.2	0.001
D2S441	9 to 16	10	11	0.324	16	0.001
			14	0.253	9	0.003

D19S433	4 to 17.2	15	13	0.251	4	0.001
			14	0.240	17	0.001
TH01	6 to 10	6	7	0.309	10	0.012
			6	0.199	9	0.143
FGA	16 to 31.2	21	23	0.167	16	0.001
			21	0.161	17	0.001
D22S1045	10 to 19	10	16	0.307	19	0.001
			15	0.303	13	0.005
D5S818	7 to 15	9	12	0.322	15	0.003
			11	0.315	14	0.025
D13S317	8 to 15	8	12	0.326	15	0.003
			11	0.302	10	0.033
D7S820	7 to 14	10	10	0.268	9.1	0.001
			11	0.234	13.1	0.001
SE33	7.3 to 35	43	18	0.109	7.3	0.001
			19	0.107	9	0.001
D10S1248	9 to 18	10	14	0.306	9	0.001
			13	0.271	18	0.001
D1S1656	10 to 19.3	16	15	0.172	13.3	0.001
			14	0.167	18	0.003
D12S391	15 to 26	16	18	0.224	19.1	0.003
			19	0.158	26	0.004
D2S1338	14 to 27	13	17	0.174	14	0.001
			19	0.159	27	0.007
X/X		2	X	0.661		
X/Y		2			Y	0.169
